# Review of the species of *Paratenetus* Spinola inhabiting America, north of Mexico (Coleoptera, Tenebrionidae)

**DOI:** 10.3897/zookeys.415.6524

**Published:** 2014-06-12

**Authors:** Yves Bousquet, Patrice Bouchard

**Affiliations:** 1 Canadian National Collection of Insects, Arachnids and Nematodes, Agriculture and Agri-Food Canada, Ottawa, Ontario, K1A 0C6, Canada

**Keywords:** Coleoptera, Tenebrionidae, North America, key

## Abstract

The North American (north of Mexico) species of the tenebrionid genus *Paratenetus* Spinola are reviewed and a key is presented for their identification. Five species are recognized, *P. gibbipennis* Motschulsky, *P. fuscus* LeConte, *P. punctatus* Spinola and two **sp. n.**, *P. exutus* [type locality: Tabusintac, Nova Scotia] and *P. texanus* [type locality: Port Isabel, Cameron County, Texas]. Two **syn. n.** are proposed: *P. cribratus* Motschulsky, 1868 with *P. gibbipennis* Motschulsky, 1868 and *P. crinitus* Fall, 1907 with *P. fuscus* LeConte, 1850. A lectotype is selected for *Paratenetus punctatus* Spinola. A type species is designated for *Storthephora* Mäklin, 1875 (*Storthephora denticollis* Mäklin, 1875).

## Introduction

The genus *Paratenetus* was proposed by Spinola in 1844 for two species, *Paratenetus lebasi* from Colombia and *Paratenetus punctatus* from the United States of America. The last mentioned species was represented by three specimens originating from the collection of Baron Dejean who received them from John Eatton LeConte. Subsequently, the genus received very little attention. In North America, John Lawrence LeConte described a new species in 1850 which he obtained during his trips to Lake Superior. In 1853–54, Victor de Motschulsky, a Russian Imperial Army Colonel, made a 10-month trip to the United States and Panama and collected at several locations including New York, Niagara Falls, Cleveland, Cincinnati, Lexington, Louisville, New Orleans, Mobile, Atlanta, Washington D.C., and Philadelphia. He described two species of *Paratenetus* in 1868 from the material he collected in Georgia. In 1907, Fall described a new species from New Mexico. Subsequently, the genus and some of its species were briefly cited in monographic works such as Blatchley (1910), Downie and Arnett (1996) and Aalbu et al. (2002a, b).

The purpose of this paper is to review the American species occurring north of Mexico and provide a key for their identification.

## Material

The study is based on the examination of about 3110 specimens borrowed from the following collections:

AFC Atlantic Forestry Centre, Fredericton, New Brunswick. Reginald P. Webster.

AMNH American Museum of Natural History, New York, New York. Lee H. Herman.

BMNH The Natural History Museum, London, United Kingdom. Maxwell Barclay.

CAS California Academy of Sciences, San Francisco, California. David H. Kavanaugh.

CMN Canadian Museum of Nature, Gatineau, Quebec. François Génier.

CNC Canadian National Collection of Insects, Archnides and Nematodes, Ottawa, Ontario.

CUIC Cornell University Insect Collection, Ithaca, New York. James K. Liebherr.

DENH Department of Entomology, University of New Hampshire. Donald S. Chandler.

ENMU Department of Biology, Eastern New Mexico University, Portales, New Mexico. Darren A. Pollock.

FSC Florida State Collection of Arthropods, Gainesville, Florida. Paul E. Skelley.

GMNH Georgia Museum of Natural History, The University of Georgia, Athens, Georgia. E. Richard Hoebeke.

GHC Gerard J. Hilchie Collection, Edmonton, Alberta.

JBWM Wallis-Roughley Museum of Entomology, University of Manitoba, Winnipeg, Manitoba. Robert E. Roughley.

JCC Janet Ciegler Collection, West Columbia, South Carolina.

LEMM Lyman Entomological Museum and Research Laboratory, McGill University, Sainte-Anne-de-Bellevue, Quebec. Stéphanie Boucher.

LSAM Louisiana State Arthropod Museum, Louisiana State University, Baton Rouge, Louisiana. Matthew L. Gimmel.

MCZ Museum of Comparative Zoology, Harvard University, Cambridge, Massachusetts. Philip Perkins.

MRSN Museo Regionale di Scienze Naturali, Torino, Italy. Luca Picciau.

NFC Northern Forestry Centre, Edmonton, Alberta. Greg R. Pohl.

RAM Royal Alberta Museum, Edmonton, Alberta. Mark Steinhilber.

RBCM Royal British Columbia Museum, Victoria, British Columbia. Claudia Copley.

RLAC Rolf L. Aalbu Collection, Sacramento, California.

RSM Royal Saskatchewan Museum, Regina, Saskatchewan. Ronald R. Hooper.

RWC Reginald P. Webster Collection, Charters Settlement, New Brunswick.

SEMC Snow Entomological Museum, University of Kansas, Lawrence, Kansas. Zachary Falin.

TAMU Texas A&M University, College Station, Texas. Edward G. Riley.

UASM Strickland Museum, University of Alberta, Edmonton, Alberta. Danny Shpeley.

UBC Spencer Entomological Museum, University of British Columbia, Vancouver, British Columbia. Karen Needham.

USNM National Museum of Natural History, Smithsonian Institute, Washington, DC. Warren E. Steiner.

ZMMU Zoological Museum, Moscow University, Moscow, Russia. Nikolay B. Nikitsky.

## Methods

The photographs were made with a Leica Digital DC500 Imaging Workstation using Zerene Stacker software and retouched with Adobe Photoshop software.

For type specimens, complete verbatim label data are given with additional information enclosed within quotation marks; individual labels are separated by a slash (/).

The distribution maps were generated using the software SimpleMappr (http://www.simplemappr.net/).

## Taxonomy

### Genus Paratenetus

Spinola, 1844

http://species-id.net/wiki/Paratenetus

Paratenetus Spinola, 1844: 116. Type species: *Paratenetus punctatus* Spinola, 1844 designated by Lucas (1920: 483).
Storthephora Mäklin, 1875: 658. Type species: *Storthephora denticollis* Mäklin, 1875 by present designation. Synonymy established by Champion (1893: 47).

#### Etymology.

Spinola (1844: 117) mentioned that the name *Paratenetus* came from a Greek adjective which supposedly means “Digne d’être observé” (worthy of being observed). The idea for the name came from the peculiar shape of the palpi and particularly the flattening of the first two labial palpomeres.

#### Description

(based on species treated only). Body short, convex, pubescent; elytra with slanting setae in addition to erect setae. Epistoma with clypeolabral membrane exposed. Eyes present, prominent. Gena not sulcate. Antenna with last three antennomeres abruptly expanded, forming a distinct, loose club. Labial palpi short, penultimate palpomere swollen, last palpomere narrow, more or less fusiform; last maxillary palpomere large, at least twice as large apically than basally. Pronotum with sides denticulate, each denticle with one or two stiff setae; surface with relatively coarse punctures. Procoxae moderately separated. Mesepimeron not closing mesocoxal cavity. Elytra without striae, with relatively coarse punctures; epipleuron distinct and relatively wide up to apex. Abdomen with distinct membrane along posterior edge of ventrites 3 and 4. Intercoxal process of first ventrite relatively wide, more or less rounded apically. Tibia not expanded apically. Metatarsomere 1 elongate, as long as next two tarsomeres combined; penultimate tarsomere deeply lobate dorsally; last tarsomere not arising at apex of penultimate tarsomere. Tarsal claw simple, not pectinate. Tarsal formula 5–5-4. Defensive glands absent.

#### Diversity.

This genus currently includes 57 species (Table 1) ranging collectively from Canada, as far north as southern Northwest Territories, south to Argentina.

**Table 1.** Checklist of *Paratenetus* species of the world.

Species	Distribution
*Paratenetus antennalis* Kulzer, 1958	Brazil
*Paratenetus atricolor* Pic, 1934	Brazil
*Paratenetus auritus* (Mäklin, 1875)	Brazil
*Paratenetus bicoloricollis* Pic, 1939	Brazil
*Paratenetus bordoni* Marcuzzi, 1994	Venezuela
*Paratenetus brevipennis* Champion, 1886	Panama
*Paratenetus cicatricosus* Motschulsky, 1868	Brazil
*Paratenetus constrictus* Champion, 1893	Mexico, Central America
*Paratenetus corticarioides* Champion, 1886	Mexico, Central America
*Paratenetus corumbanus* Pic, 1934	Brazil
*Paratenetus crenulatus* Champion, 1886	Panama
*Paratenetus denticollis* (Mäklin, 1875)	Venezuela
*Paratenetus denticulatus* Champion, 1886	Panama
*Paratenetus discoidalis* Pic, 1939	Brazil
*Paratenetus donckieri* Pic, 1925	Brazil
*Paratenetus ensellatus* Pic, 1934	Brazil
*Paratenetus epitragoides* Berg, 1889	Argentina
*Paratenetus exutus* Bousquet & Bouchard, sp. n.	Canada, U.S.A.
*Paratenetus foveithorax* Ferrer & Ødegaard, 2005	Panama
*Paratenetus freyi* Kulzer, 1958	Brazil
*Paratenetus fuscus* LeConte, 1850	Canada, U.S.A.
*Paratenetus germaini* Pic, 1926	Bolivia
*Paratenetus gibbipennis* Motschulsky, 1868	Canada, U.S.A.
*Paratenetus gounellei* Pic, 1920	Brazil
*Paratenetus grandicornis* Motschulsky, 1868	Nicaragua, Panama
*Paratenetus grandis* Pic, 1920	Brazil
*Paratenetus helgae* Kulzer, 1958	Trinidad
*Paratenetus huequensis* Marcuzzi, 1994	Venezuela
*Paratenetus humeralis* Pic, 1934	Brazil
*Paratenetus inaequalis* Pic, 1925	Brazil
*Paratenetus inermis* Champion, 1893	Guatemala
*Paratenetus koltzei* Pic, 1939	Mexico
*Paratenetus laticollis* Pic, 1925	Brazil
*Paratenetus latipennis* Pic, 1920	Peru
*Paratenetus lebasi* Spinola, 1844	Columbia
*Paratenetus limbaticollis* Pic, 1921	Brazil
*Paratenetus lithophiloides* Pic, 1921	Brazil
*Paratenetus longicornis* Pic, 1925	Guadeloupe
*Paratenetus luridus* Motschulsky, 1868	Brazil
*Paratenetus mexicanus* Pic, 1925	Mexico
*Paratenetus nigricornis* Champion, 1893	Mexico, Central America
*Paratenetus obovatus* Champion, 1893	Central America
*Paratenetus punctatus* Spinola, 1844	Canada, U.S.A.
*Paratenetus punctulatus* Champion, 1893	Mexico, Central America
*Paratenetus ruficornis* Champion, 1886	Panama
*Paratenetus sexdentatus* Champion, 1893	Central America
*Paratenetus sparsepunctatus* Pic, 1939	Argentina
*Paratenetus suturalis* Pic, 1921	Brazil
*Paratenetus testaceicornis* Pic, 1925	Brazil
*Paratenetus testaceipes* Pic, 1934	Bolivia
*Paratenetus testaceus* Pic, 1920	Costa Rica
*Paratenetus texanus* Bousquet & Bouchard, n.sp.	U.S.A., Mexico
*Paratenetus tibialis* Champion, 1893	Mexico, Central America
*Paratenetus tropicalis* Motschulsky, 1868	Mexico, Central America
*Paratenetus tuberculatus* Champion, 1886	Panama
*Paratenetus vianai* Pic, 1939	Argentina
*Paratenetus villosus* Champion, 1886	Mexico, Central America

#### Taxonomic position.

Spinola (1844) originally placed *Paratenetus* in his Clérites Corynétoïdes (currently Cleridae: Korynetinae). Agassiz (1846: 119) listed it in the family “Tenebrionites.” Melsheimer (1853: 45) transferred the genus to the family Cryptophagidae. LeConte (1862: 232) moved *Paratenetus* back in the family Tenebrionidae, and placed it in the tribe Heterotarsini, a position that was followed by several authors including Horn (1870: 373), Gebien (1911: 471), Leng (1920: 236), Gebien (1941: 821) and Arnett (1962: 688). In 1918, Leng mentioned that “the genera *Paratenetus*, *Prataeus* and *Anaedus* seem to be near the Lagriidae on account of the similarity in their larval stages” and Böving and Craighead (1931: 42) moved the genera of Heterotarsini (except *Heterotarsus* Latreille) from the tenebrionids to the lagriids based also on the morphology of the larvae. The study of the ovipositor structures by Tschinkel and Doyen (1980: 367) supported also the position of *Paratenetus* within the subfamily Lagriinae rather than the subfamily Tenebrioninae. Ardoin (1961: 33) placed the genera of Heterotarsini (except *Heterotarsus*) in the lagriine subtribe Lupropina of the tribe Adeliini. Parsons (1976: 211) listed *Paratenetus* in the lagriid subfamily Lupropinae. Doyen and Tschinkel (1982: 183) indicated that the genus may belong to the belopines, currently a valid lagriine tribe. Campbell (1991: 261) listed *Paratenetus* in the lagriid subfamily Goniaderinae and Aalbu et al. (2002a: 509; 2002b: 484) retained also the genus in the lagriine tribe Goniaderini. On the other hand, Ferrer and Ødegaard (2005: 648) included it in the lagriine tribe Lupropini following Ardoin (1961) and Parsons (1976).

We did not investigate the taxonomic position of the genus *Paratenetus* but we accept, following Aalbu et al. (2002a; 2002b), its placement in the tribe Goniaderini of the subfamily Lagriinae within the Tenebrionidae.

#### Biology.

The biology of members of *Paratenetus* is poorly known. Many of the specimens seen in this study were collected in leaf litter in forested areas or in nests of the tent caterpillar genus *Malacosoma* (Lepidoptera: Lasiocampidae). All three winged species have been collected at black light. Steiner (1995: 508) commented that *Paratenetus* species pupate on the inner surfaces of rolled dead leaves (in which the larvae live) either hanging on fallen tree branches or on the ground.

#### Notes.

There are two types of setae on the elytra of *Paratenetus*: erect and slanting. The slanting setae are characterized as subdepressed when the angle between the base of the seta and the elytra is between 10 and 40°, semierect when the angle is between 40 and 60°, and suberect when the angle is between 60 and 80°.

#### Key to North American (north of Mexico) species of *Paratenetus*

**Table:** 

1	Metaventrite short, length along midline subequal to or shorter than length of abdominal ventrite 2 along midline	2
–	Metaventrite longer, length along midline longer than length of abdominal ventrite 2 along midline	3
2	Elytra with very few, short erect setae	*Paratenetus gibbipennis* Motschulsky
–	Elytra with numerous, long erect setae	*Paratenetus fuscus* LeConte
3	Antennomere 8 transverse. Metaventrite quite distinctly darker than first two abdominal ventrites in the vast majority of specimens, not or only slightly darker in a few specimens. Protibia of male without calcar	*Paratenetus exutus* Bousquet & Bouchard
–	Antennomere 8 subquadrate or slightly elongate. Metaventrite not darker than first two abdominal ventrites in the vast majority of specimens, slightly darker in a few specimens. Protibia of male with calcar	4
4	Pronotum with maximum width anterior of midlength (Fig. 3); punctures narrowly spaced, in part subcontiguous over lateral half [widely distributed in eastern North America]	*Paratenetus punctatus* Spinola
–	Pronotum with maximum width at midlength (Fig. 4); punctures moderately dense, not subcontiguous even over lateral half [known only from Texas, Louisiana and Florida in North America]	*Paratenetus texanus* Bousquet & Bouchard

### Paratenetus gibbipennis

Motschulsky, 1868

http://species-id.net/wiki/Paratenetus_gibbipennis

Figs 5 , 10

Paratenetus gibbipennis Motschulsky, 1868: 193. Type locality: «Atlanta, Géorgie américaine» (original citation).
Paratenetus cribratus Motschulsky, 1868: 193. Type locality: «Géorgie américaine» (original citation). syn. n.

#### Type material.

Motschulsky’s collection at ZMMU contains a single specimen, a female, under the name *Paratenetus gibbipennis*. It bears the following labels: “[green round disc] / [small brick red square label] / type [handwritten] / Paratenetus gibbipennis Motch Am. b. Mobile [handwritten on a rectangular green label].” The specimen is intact although many of the setae on the pronotum and elytra are gone. The provenance of the specimen is doubtful. In the key to the *Paratenetus* in his collection, Motschulsky (1868: 193) mentioned that the species was collected in Atlanta but Mobile is listed on one of the type labels. Motschulsky collected in both localities during his 10-month trip to America in 1853–54.

Motschulsky’s collection contains a single specimen, a male, under the name *Paratenetus cribratus*. It bears the following labels: “[green round disc] / Atlanta [handwritten] / type [handwritten] / Paratenetus cribratus Motch Am. bor. Atlanta [handwritten on a rectangular green label].” The specimen is missing the left antennomeres 3–11 and the posterior legs.

#### Note about synonymy.

Motschulsky (1868) separated *Paratenetus gibbipennis* and *Paratenetus cribratus* on the account that the first species has the lateral denticles of the pronotum very short while the second species has strong denticles. From an examination of the types, we cannot sustain Motschulsky’s affirmation; the denticles are basically of the same size on both specimens.

#### Diagnosis.

This species and *Paratenetus fuscus* differ from the other three species treated by having the metaventrite very short. *Paratenetus gibbipennis* differs from *Paratenetus fuscus* by having few short erect setae on the elytra.

#### Description.

Body dorsally reddish yellow to dark reddish brown, legs paler, yellow to reddish yellow; antennal club not darkened in most specimens; metaventrite not darker than first two abdominal ventrites. Antennomere 8 subquadrate or very slightly transverse. Pronotum with maximum width near midlength or slightly anterior to midlength; punctures moderately dense, not subcontiguous even over lateral half. Elytra very convex; slanting setae subdepressed, erect setae very few, short. Metaventrite short, length along midline clearly shorter than length of abdominal ventrite 2 along midline. Male protibia with calcar near middle along ventral surface; male mesotibia with short, in some specimens very short, more or less perpendicular preapical protuberance. Parameres with sides more or less parallel towards apex, apex not particularly acute (Fig. 5).

Length: 2.5–3.2 mm.

#### Distribution.

This species ranges from southern Maine to southwestern Manitoba, south to central Texas, southwestern Alabama, and central South Carolina (Fig. 10).

#### Records.

We have seen 660 specimens from the following localities. Canada. **Manitoba.** “Tp.2, Rge. 15, E. 1 Mer.” (CNC). Rennie (CNC). Brandon (RBCM). Telford (NFC). Winnipeg (RBCM). **Ontario.** “Jonction Hwy 17 & 71” (CNC). Bainsville (LEMM). Prince Edward Co. (CNC, CUIC, MCZ, CAS, USNM). Lancaster (LEMM). Chaffeys Locks Biol. Station (CNC). Alfred (CNC). Long Sault (CNC). 10 km W North Gower (CNC). Nepean (CNC). Belleville (CUIC). Thwartway Island, St. Lawrence Is. Nat. Park (CNC). Point Pelee (CNC). 2 km SE Spencerville (CNC). 4 km SW Kanata (CNC). Ottawa (CNC). Constance Bay (CMN). 4 km N of Westport (CNC). Campden (CNC). Rondeau Prov. Park (CNC). Arnprior (CNC). Erieau (CNC). 7 km W. Carleton Place (CNC). Blackburn (CNC). Normandale (CNC). Pelee Island (CNC). Hamilton (CNC). Flint Hill, nr Kemptville (CNC). Trenton (CNC). Toronto (CUIC, MCZ, USNM). Milton (CNC). **Quebec.** Montreal (CNC). Rigaud (CNC). Gatineau (CNC, CAS). Blind Lake, Gatineau Park (CNC). Gatineau Park (DENH). Hudson Heights (CNC). Oka (CNC). Ormstown (CNC). United States of America. **Alabama.** *Mobile Co.*: Mt. Vernon (CUIC). *Monroe Co.*: Haines Island Park, 3.5 mi. W Franklin (FSC). **Connecticut.** *Fairfield Co.*: Westport (AMNH). *Litchfield Co.*: Torrington (DENH); Canaan (TAMU); Cornwall (CUIC, AMNH). **Georgia.** *Clarke Co.*: 5 mi W Athens (GMNH); Whitehall Forest (GMNH). *Rabun Co.*: Tally Mill Crk. at Hwy 28 (CNC); Satolah (CNC). **Illinois.** “N. Ill.” (MCZ). “Ill.” (USNM). **Maine.** *Cumberland Co.*: Portland (CNC). *Kennebec Co.*: Monmouth (MCZ). *Oxford Co.*: Paris (MCZ). **Massachusetts.** “Mass.” (USNM). *Bristol Co.*: Swansea (MCZ); Somerset (MCZ); Dighton (MCZ); Fall River (MCZ). *Hampshire Co.*: Mount Tom (MCZ). *Middlesex Co.*: Waverly (USNM); Framingham (CNC, CUIC, MCZ); Lowell (MCZ); Sherborn (CUIC, AMNH); Hopkinton (CUIC); Cambridge (MCZ, USNM); Newton (MCZ). *Norfolk Co.*: Brookline (CUIC, MCZ, AMNH); Sharon (CUIC). **Michigan.** *Marquette Co.*: Marquette (USNM). *Oakland Co.* (CUIC). *Wayne Co.*: Detroit (MCZ, USNM). **Minnesota.** *Crow Wing Co.*: Brainerd (CNC). *Hennepin Co.*: Lake Minnetonka (CUIC). **Missouri.** *Saint Charles Co.*: St. Charles (MCZ). **Nebraska.** *Cuming Co.*: West Point (USNM). **New Hampshire.** *Grafton Co.*: Franconia (MCZ, AMNH); 0.5 mi S Rumney (DENH); Hanover (DENH). *Rockingham Co.*: Hampton (DENH, USNM); Odiorne Point State Park (DENH). *Strafford Co.*: Somersworth (DENH); Durham (DENH); 3 mi. E Durham (DENH). **New Jersey.** *Union Co.*: Union (CUIC, AMNH); Roselle (USNM); Elizabeth (USNM). **New York.** “S[taten] I[sland]” (MCZ). *Dutchess Co.*: Bulls Head (AMNH). *Oswego Co.*: North Pond (CUIC); Oswego (CUIC). *Queens Co.*: Flushing, L.I. (CUIC). **North Carolina.** *Haywood Co.*: Round Knob (USNM). *Henderson Co.*: 14 mi NW Hendersonville (SEMC). *Macon Co.*: 3 mi NW Highlands (DENH). *Montgomery Co.*: 2 mi S Eldorado (DENH). *Yancey Co.*: Black Mountains (AMNH). **North Dakota.** *Richland Co.*: Mirror Pool (USNM). **Ohio.** “Ohio” (MCZ). **Pennsylvania.** “Penn” (MCZ). *Allegheny Co.*: Allegheny (USNM). **Rhode Island.** “R.I.” (USNM). **South Carolina.** *Calhoun Co.*: Wannamaker NP, St. Matthews (JCC). *Chester Co.*: Leeds (JCC). *Edgefield Co.*: Sumter Nat. For. (DENH). *Lexington Co.*: West Columbia (JCC). *Newberry Co.*: Billy Dreher Island State Park (JCC). *Pickens Co.*: Nine Times (JCC). *Union Co.*: Sedalia (JCC). **Tennessee.** “Chilhowee Mountain” (CMN). *Blount Co.*: Rt. 129 just below rd. at The Narrows Overlook, GSMNP (LSAM); Ace Gap, GSMNP (LSAM). *Cocke Co.*: Gabes Mtn., GSMNP (LSAM). *Sevier Co.*: 0.5 mi W Laurel Falls Trailhead, GSMNP (LSAM); Twin Creeks, GSMNP (LSAM); Grapeyard Ridge (LSAM). **Texas.** *Blanco Co.*: Cypress Mill (USNM). **Virginia.** *Giles Co.*: Bald Knob, Mountain Lake (USNM); 9 km N Mountain Lake (USNM). *Lee Co.*: Pennington Gap (USNM). **Wisconsin.** *Bayfield Co.*: Bayfield (USNM). *Dane Co.*: Madison (TAMU). *Shawano Co.*: Tilleda (FSC).

#### Remarks.

Females are much more abundant in collections than males. Of 183 specimens randomly selected, 8 were males (4.4%) and 175 were females (95.6%). The males came from Georgia (n=1), Alabama (n=6), and Missouri (n=1). No males were found among the 160+ randomly selected specimens from Canada and the northern states.

Specimens were collected in January (n=1), February (n=1), March (n=89), April (n=64), May (n=8), June (n=61), July (n=20), August (n=95), September (n=31), October (n=38), November (n=6) and December (n=2).

Labels on specimens read “in leaf litter” (6 specimens); “in leaf litter of black birch and shrubs around and on areas of exposed rock” (71); “forest litter sifting” (2); “forest litter” (3); “moist forest berlese” (1).

### Paratenetus fuscus

LeConte, 1850

http://species-id.net/wiki/Paratenetus_fuscus

Figs 6 , 11

Paratenetus fuscus LeConte, 1850: 223. Type locality: Lake Superior (inferred from the title of the book).
Paratenetus crinitus Fall, 1907: 253. Type locality: «Trout Spring [New Mexico]» (original citation). syn. n.

#### Type material.

LeConte’s collection at MCZ contains a single male specimen under the name *Paratenetus fuscus*. It bears the following labels: “[pale green round disc] / Type 4684 [partially handwritten on a red square label] / P. fuscus Lec. [handwritten].” The specimen is intact.

Fall described *Paratenetus crinitus* from one specimen now at the MCZ. It bears the following labels: “Trout sp. N.M. May [handwritten] / crinitus. Type [partially handwritten] / M.C.Z Type 24612 [red square label] / H.C. Fall Collection.” The specimen is intact.

#### Note about synonymy.

Fall (1907: 253) described his *Paratenetus crinitus* and mentioned that “in *crinitus* the metasternum is almost as short as in *fuscus*, which species is, however, very distinct by its subinflated elytra, more rounded sides of the prothorax and absence of erect hairs on the upper surface.” Obviously Fall did not study the syntype in LeConte’s collection since the specimen bears many erect hairs. LeConte never mentioned that character in his description and obviously Fall misidentified LeConte’s species. We have studied the type specimens of both species and find no structural differences to separate them.

#### Diagnosis.

This species differs from *Paratenetus gibbipennis* by the character states listed in the description.

#### Description.

Same character states as *Paratenetus gibbipennis* except for the following: slanting setae on elytra less depressed, semierect, occasionally even suberect; erect setae numerous, in seven or eight rows; metaventrite slightly longer, length along midline subequal to slightly shorter than length of abdominal ventrite 2 along midline.

#### Distribution.

This species ranges from Quebec City to the Rocky Mountains in northeastern British Columbia, north to southern Northwest Territories, south to northern New Mexico, northeastern Kansas, and Maryland (Fig. 11).

#### Records.

We have seen 305 specimens from the following localities. Canada. **Alberta.** “Tp. 74, Rge. 25, W. 5 Mer.” (CNC). “Tp. 11, Rge. 1, W. 5 Mer.” (CNC). Waterton Park (CNC). Calgary (CNC, ENMU, CAS, GHC). Castor (UASM). Edmonton (CUIC, UASM). Stettler (CNC). Cochrane (CNC). 30 km W Cochrane (CNC). Cypress Hills (CNC). Waiparous (CNC). Jumpingpound Creek (CNC). Sundre (CNC). Elkwater (CNC). **British Columbia.** North Pine (UBC). Pouce Coupe (UBC). **Manitoba.** “Tp. 9, Rge. 16, W. 1 Mer.” (CNC). Aweme (CNC, JBWM, RAM). Brandon (RBCM). Sandilands (JBWM). Birds Hill Prov. Park (ENMU). Husavik (CNC). **Northwest Territories.** Louise Falls, Hay River (CNC). Simpson (CAS). **Ontario.** Prince Edward Co. (CNC, USNM). Pelee Island (CNC). Ottawa (CNC). Constance Bay (CMN). Trenton (CNC). Arnprior (CNC). **Quebec.** Chelsea (CNC). Rigaud (CNC). Sainte-Croix-de-Lotbiniere (LEMM). Cap Rouge (CNC). **Saskatchewan.** Lac La Ronge (CNC). Morse (RSM). Rosefield (RSM). Oxbow (USNM). United States of America. **Colorado.** *Boulder Co.*: Boulder (CNC, USNM). *Custer Co.* (USNM). *Douglas Co.*: Castle Rock (CNC). *El Paso Co.*: Colorado Springs (USNM). *Jefferson Co.*: Lookout Mountain (CUIC). **Connecticut.** *Fairfield Co.*: Westport (AMNH). **District of Columbia.** “DC” (CNC, USNM). **Illinois.** “N. Ill.” (MCZ). *Champaign Co.*: Urbana (USNM). **Iowa.** “Iowa” (MCZ, AMNH). *Johnson Co.*: Iowa City (USNM, AMNH). *Story Co.*: Ames (USNM). **Kansas.** “Ks” (USNM). “Kans” (USNM). *Douglas Co.*: Lawrence (CNC). *Shawnee Co.*: Topeka (USNM). **Maryland.** “Md.” (CNC). *Anne Arundel Co.*: 6 km ESE Laurel (USNM). **Massachusetts.** “Mass” (USNM). *Essex Co.*: Lynn (MCZ, USNM); Salem (USNM). *Middlesex Co.*: Sherborn (MCZ, USNM); Tyngsboro (MCZ). *Norfolk Co.*: Brookline (MCZ). **Michigan.** *Marquette Co.*: Marquette (USNM). **Montana.** “Mont.” (CUIC). “Montana” (USNM). *Dawson Co.*: Glendive (USNM). *Powder River Co.*: Fort Howes (USNM). *Rosebud Co.*: Colstrip (USNM). **Nebraska.** “Neb.” (USNM). *Red Willow Co.*: McCook (MCZ). **New Mexico.** “N. Mex.” (USNM). *San Miguel Co.*: Trout Spring (MCZ). **New York.** “N.Y.” (CUIC, MCZ). *Westchester Co.*: Peekskill (CUIC). *New York Co.*: Central Park, L.I. (USNM). **North Dakota.** *Richland Co.*: Mirror Pool (USNM). **Rhode Island.** “R.I.” (USNM). **South Dakota.** *Jackson Co.*: Cottonwood (RLAC). **Tennessee.** “Tenn.” (MCZ). **Vermont.** “Vt.” (MCZ). **Wisconsin.** “Wis” (MCZ). *Sauk Co.*: Spring Green (USNM). **Wyoming.** *Converse Co.*: 11 mi N Douglas (CNC). *Laramie Co.*: Cheyenne (USNM).

#### Remarks.

Females are a little more common in collections than males. Of 45 randomly selected specimens, 28 (62%) were females and 17 (38%) were males.

Specimens were collected in February (n=1), March (n=30), April (n=58), May (n=8), June (n=40), July (n=7), August (n=19), September (n=14), October (n=5) and November (n=4).

### Paratenetus punctatus

Spinola, 1844

http://species-id.net/wiki/Paratenetus_punctatus

Figs 3 , 8 , 12

Latridius pubescens Say, 1826: 265 [*nomen dubium*]. Type locality: United States (inferred from title of the paper).
Paratenetus punctatus Spinola, 1844: 118. Type locality: «Etats-unis de l’Amérique septentrionale» (original citation). Synonymy established by LeConte (1859: 325).

#### Type material.

Most of Say’s entomological collection has been destroyed and we are unaware that a syntype of his *Latridius pubescens* survived. LeConte (1859: 325) based his interpretation of Say’s species on the original description. For nomenclatural stability, we believe it is best to consider *Latridius pubescens* Say as a *nomen dubium* and retain the long accepted name *Paratenetus punctatus* Spinola for this species.

Spinola (1844: 119) indicated that he had three specimens of *Paratenetus punctatus* which came from Dejean’s collection. These specimens were received for study from the Museo Regionale di Scienze Naturali in Turin (MRSN). The first specimen, probably a female, is labeled “Paratenetus punctatus Ekis 1974 [handwritten]”; the second, a male “Paralectotype Paratenetus punctatus Spinola Ekis 74 [handwritten]”; and the third, a female “Lectotype Paratenetus punctatus (Spinola) Ekis 74 [handwritten]”. The first two specimens correspond neither to our concept of *Paratenetus punctatus* nor to any other North American species we have seen. The specimens are in poor condition, with almost all the setae gone, but they appear to be conspecific. Although Spinola indicated that all three of his specimens came from the United States and were provided by “Mr. [John Eatton] LeConte,” these two specimens may have originated from Mexico, Central America or South America. The third specimen, a small individual (3.2 mm), fits our concept of *Paratenetus punctatus* and is here selected as lectotype. The label “Lectotype Paratenetus punctatus Spinola des. Y. Bousquet 2012” has been attached to the specimen.

#### Diagnosis.

Many specimens of *Paratenetus punctatus* can be separated from the other North American species of *Paratenetus* by their large size (3 mm or more). The vast majority of specimens of the other species are less than 3 mm long. Otherwise, the species can be separated from *Paratenetus exutus* in having the antennomere 8 subquadrate, the pronotum wider clearly anterior to the midlength, the punctation on the pronotum coarser, the slanting setae on the elytra slightly longer and more erect and the protibia of the male with a calcar along ventral surface. From *Paratenetus texanus*, this species is best separated in having the pronotum widest anterior to the midlength and the punctures on the pronotum coarser and denser, in part subcontiguous along the lateral half.

#### Description.

Body dorsally uniformly pale to dark reddish brown in most specimens, with the pronotum and head slightly darker than elytra and legs in some specimens; antennal club darker than antennomeres 1–8; metaventrite not darker than first two abdominal ventrites in the vast majority of specimens, slightly darker in a few specimens. Antennomere 8 subquadrate. Pronotum with maximum width anterior of midlength (Fig. 3); punctures narrowly spaced, in part subcontiguous over lateral half. Elytra less convex than for *Paratenetus gibbipennis* and *Paratenetus fuscus*; slanting setae semierect in the vast majority of specimens, suberect in some specimens, erect setae few. Metaventrite long, length along midline longer than length of abdominal ventrite 2 along midline. Male protibia with calcar near middle along ventral surface; male mesotibia with very short, preapical spine, oriented perpendicularly or obliquely to long axis of tibia. Parameres with sides more or less parallel to very slightly convergent towards apex; apex more or less rounded (Fig. 8).

Length: 3.0–4.0 mm.

#### Distribution.

This species ranges from New Brunswick to southeastern Manitoba, south to eastern Texas, southern Mississippi, and southeastern Florida (Fig. 12).

#### Records.

We have seen 1215 specimens from the following localities. Canada. **Manitoba.** “Tp. 3, Rge. 17, E.1 Mer.” (CNC). Victoria Beach (JBWM). **New Brunswick.** Jackson Falls, Carleton Co. (RWC). 10 km NW New River Beach, Charlotte Co. (AFC). 12 km SSE Upper Napan, Northumberland Co. (RWC). Cranberry Lake Protected Natural Area, Queens Co. (AFC). Acadia Research Forest, Sunbury Co. (AFC, RWC). Charters Settlement, York Co. (RWC). Canterbury, York Co. (RWC). 15 km W Tracy, York Co. (RWC). 14 km WSW Tracy, York Co. (AFC). **Ontario.** Ottawa (CNC). Constance Bay (CMN). Flint Hill, nr Kemptville (CNC). Ad & Lennox Co. (CNC, FSC, AMNH). Pelee Island (CNC). Leamington (CNC). Hastings Co. (CNC). Walsingham (CNC). Prince Edward Co. (CNC, USNM). Point Pelee (CNC). Rondeau Prov. Pk. (CNC). Arnprior (CNC). Chaffeys Locks (CNC). Northumberland Co. (CNC). Sudbury (CNC). Hamilton (CNC). Gordon Island (St. Lawrence Is. Nat. Pk.) (CNC). 13 km W of Mattawa (CNC). Toronto (CUIC). 2–5 km W Mallorytown Landing (CMN). **Quebec.** Rouville (CNC). Fort Coulonge (CNC). Parc Provincial d’Oka (CNC). Berthierville (CNC). Parc de la Gatineau (CNC). Laniel (CNC). Ile-du-Grand-Calumet (Pontiac) (CNC). Rigaud (CNC). Montreal (CNC). Parc de la Yamaska (CNC). United States of America. **Arkansas.** *Garland Co.*: 3 mi W Crystal Springs (SEMC). **Connecticut.** *Fairfield Co.*: Westport (AMNH). *Litchfield Co.*: Litchfield (AMNH). *New Haven Co.*: Middlebury (USNM); Hamden (CUIC). **District of Columbia.** “D.Col.” (USNM). Takoma Park (USNM). Washington (USNM). **Florida.** “Fla” (USNM). *Alachua Co.*: Gainesville (FSC); Newnans Lake (RLAC). *Duval Co.*: Jacksonville (USNM). *Escambia Co.*: Pensacola (FSC). *Hillsborough Co.*: Tampa (USNM). *Indian River Co.*: south of Vero Beach (FSC). *Levy Co.*: 4 mi SW Archer (FSC). *Marion Co.*: Juniper Springs (FSC); Rainbow Springs (FSC); Ocala National Forest (CMN, FSC). *Palm Beach Co.*: Lake Worth (CUIC). *Polk Co.*: Lake Marion Creek (GMNH). *Putnam Co.*: 2.5 mi NE Florahome (FSC); Crescent City (USNM); Welaka Exp. Sta. (DENH, LSAM). *Saint Johns Co.*: St. Augustine (MCZ). *Santa Rosa Co.*: 4 mi N Munson (LSAM). *Seminole Co.*: Lake Mary (MCZ). *Volusia Co.*: South Daytona (CNC); Daytona (USNM). **Georgia.** *Charlton Co.*: 2.8 mi N Saint George (FSC). *Johnson Co.*: 1 mi E Kite (FSC); Suwanee Canal Rec. Area (FSC). *Montgomery Co.*: 5 mi W Uvalda (GMNH). *Rabun Co.* (MCZ); Clayton (MCZ). *Tattnall Co.* (FSC). *Union Co.*: “Herbert Reece Park” (GMNH). **Illinois.** *Knox Co.*: Galesburgh (MCZ). *Macon Co.* (FSC). **Indiana.** *Allen Co.*: “Schoaf Park” (USNM). *Howard Co.*: “NW Howard Co.” (LSAM). *Jasper Co.*: Jasper/Pulaski St. Forest (USNM). *LaPorte Co.*: Michigan City (USNM). *Monroe Co.*: Bloomington (FSC, USNM). *Porter Co.*: Dunes St. Pk. (RLAC). *Tippecanoe Co.* (CUIC, USNM, AMNH); “McCormick Woods” (USNM). **Iowa.** “Iowa” (USNM). *Johnson Co.*: Iowa City (USNM). *Polk Co.*: “Brown WDS Psv” (CUIC); W. Saylorville Lake (CUIC, USNM). **Kansas.** “Kans” (USNM). *Cherokee Co.*: 2 mi S Galena (SEMC). *Crawford Co.*: Pittsburg (SEMC); 2 mi W Pittsburg (SEMC). *Douglas Co.*: 2 mi NW of Baldwin (SEMC). *Jackson Co.*: 6.5 km W Mayetta (SEMC). *Jefferson Co.*: 1 km SW Perry State Park (SEMC). *Johnson Co.*: Overland Park Arboretum (SEMC). *Labette Co.*: Big Hill Reservoir (SEMC). *Neosho Co.*: 2 mi SE Erie (SEMC). *Sedgwick Co.*: 0.5 mi S of Derby (SEMC). *Shawnee Co.*: S of intersection Woodring Rd & 69th St (SEMC). **Kentucky.** “Ky” (USNM). *Marshall Co.* (FSC). **Louisiana.** *Caddo Parish*: Jacobs Nature Park (LSAM). *Claiborne Parish*: Corney Lake (CNC). *East Feliciana Parish*: Idlewild Exp. Station (LSAM). *Livingston Parish*: Livingston (LSAM). *Natchitoches Parish*: Kisatchie Nat. For. (LSAM). *West Feliciana Parish*: Saint Francisville (CMN); Tunica Hills, 0.5 mi W Weyanoke (LSAM). **Maine.** *Androscoggin Co.*: Wales (MCZ). *Cumberland Co.*: Casco (CUIC). *Franklin Co.*: Dead River (USNM); Farmington (USNM). *Kennebec Co.*: Augusta (DENH); Monmouth (MCZ). *Oxford Co.*: Rumford (USNM); Bethel (AMNH). *Penobscot Co.*: Lee (DENH); Passadumkeag (CUIC). *Piscataquis Co.*: Greenville (USNM). *Washington Co.*: Beddington (USNM). *York Co.*: West Lebanon (DENH). **Maryland.** *Allegany Co.*: Piclic Ridge, 5 km SE Pratt (USNM); Fifteen Mile Creek (RLAC). *Anne Arundel Co.*: 8 km ESE Laurel (USNM); 6 km ESE Laurel (USNM); Edgewater (USNM); 6 km S Edgewater (USNM); 3 km WSW Bristol at Jug Bay (USNM); Odenton (CUIC, USNM). *Baltimore Co.*: 4 km SW Cockeysville (USNM); Catonsville (USNM). *Calvert Co.*: Plum Point (USNM). *Carroll Co.*: Eldersburgh (USNM). *Cecil Co.*: Pleasant Hill (USNM); Port Deposit (USNM). *Frederick Co.*: 2 mi W Thurmont (USNM). *Garrett Co.*: Rock Lodge, 4 km SW Bittinger (USNM); 7 mi N Oakland (USNM). *Montgomery Co.*: Kensington (USNM); Potomac (USNM); Rockville (USNM); Plummers Island (USNM); Great Falls (USNM); Hughes Hollow area, 5 km W Seneca (USNM). *Prince Georges Co.*: Cheverly (USNM); Bladensburg (USNM); Takoma Park (USNM); Laurel (USNM); Priest Bridge (USNM); Oxon Hill (USNM); Beltsville (USNM); Greenbelt (Park) (USNM); Bowie (USNM). *Somerset Co.*: Shelltown (USNM). *Talbot Co.*: 3 km SE Easton (USNM); Wittman (USNM); McDaniel (USNM). *Worcester Co.*: Assateague Island (USNM). **Massachusetts.** *Barnstable Co.*: Cape Cod (CNC). *Bristol Co.*: Dartmouth (MCZ). *Essex Co.*: Nahant (MCZ). *Hampden Co.*: Springfield (USNM). *Middlesex Co.*: Lincoln (MCZ); Sherborn (MCZ); Framingham (MCZ); Hopkinton (MCZ); Tyngsboro (MCZ); Natick (MCZ); Cambridge (MCZ). *Norfolk Co.*: Brookline (MCZ). *Plymouth Co.*: Marion (USNM). *Suffolk Co.*: Boston (MCZ); Jamaica Plain (CUIC). **Michigan.** *Shiawassee Co.*: Rose Lake Wldlf. Exp. Station (USNM). *Wayne Co.*: Detroit (USNM). **Minnesota.** *Hennepin Co.*: Minneapolis (CNC). *Houston Co.*: Winnebago Cr. Vy., 3–4 m NE Eitzen (USNM). *Saint Louis Co.*: Duluth (MCZ). **Mississippi.** *George Co.*: Lucedale (CUIC). *Greene Co.*: Leakesville (CUIC). *Lauderdale Co.*: Marion (MCZ). **Missouri.** *Barry Co.*: Mark Twain Nat. For. (FSC). *Boone Co.*: Ashland Wildlife Ar. (TAMU). *Clay Co.*: Cooley Lake (FSC). *Greene Co.*: near James River (TAMU). *Jackson Co.*: Raytown (FSC). *Oregon Co.*: Mark Twain Nat. For. (FSC). *Randolph Co.*: 1 mi E Moberly (TAMU). **New Hampshire.** “N.H.” (USNM). *Coos Co.*: Gorham (CNC); Mt. Washington (DENH, MCZ, AMNH). *Grafton Co.*: Mt. Moosilauke (MCZ); Bedell Bridge St. Pk. (DENH); Bath (DENH). *Hillsborough Co.*: Antrim (MCZ). *Merrimack Co.*: Concord (DENH). *Strafford Co.*: 1 mi SW Durham (DENH); Dover (DENH). **New Jersey.** “N.J.” (AMNH). *Atlantic Co.*: Buena (MCZ). *Bergen Co.*: Fort Lee (AMNH). *Burlington Co.*: Wharton State Forest (TAMU); Pemberton (USNM); 7 mi E Batsto (USNM). *Cumberland Co.*: Rutgers Exp. Sta. (USNM). *Essex Co.*: South Orange (MCZ); Eagle Rock (USNM); Montclair (USNM). *Gloucester Co.*: Malaga (USNM). *Monmouth Co.*: Highlands (USNM). *Monroe Co.*: Delaware Water Gap (USNM). *Morris Co.*: Boonton (USNM). *Ocean Co.*: Lakehurst (CUIC, CUIC, USNM). *Orange Co.*: Greenwood Lake (CUIC, USNM). **New York.** “S.I.” (USNM). *Albany Co.*: Delmar (CUIC); Rensselaerville (USNM). *Clinton Co.*: vic. Taylor Pond Campground (GMNH). *Erie Co.*: Buffalo (MCZ, USNM). *Essex Co.*: New Russia (CUIC); Whiteface Mt. (USNM). *Orange Co.*: Greenwood Lake (CUIC); Fort Montgomery (CUIC); West Point (USNM). *Putnam Co.*: Brewster (CUIC). *Rockland Co.*: Nyack (CUIC). *Saint Lawrence Co.*: Rossie (USNM). *Seneca Co.*: Willard (USNM). *Suffolk Co.*: Huntington, Long Island (DENH); Southold, L.I. (CUIC); Wyandanch, L.I. (USNM); Bellport (USNM); Yaphank (USNM). *Tompkins Co.*: Ithaca (CUIC, FSC, USNM); Dryden (CUIC). *Yates Co.*: Seneca Lake (USNM). **North Carolina.** “N.C.” (MCZ). “Round Knob” (USNM). *Brunswick Co.*: Southport (FSC). *Buncombe Co.*: 6 mi S Asheville (SEMC). *Burke Co.*: Linville Falls (CNC). *Columbus Co.*: Lake Waccamaw (USNM). *Gates Co.*: 6 km ENE Corapeake (USNM). *Haywood Co.*: Cove Creek (JCC); Cataloochee Divide (LSAM, MCZ); 9 mi W Waynesville (SEMC). *Henderson Co.*: Fletcher (FSC). *Jackson Co.*: Balsam (USNM). *Macon Co.*: Nantahala Gap (CUIC); Highlands (CMN, CNC). *Mitchell Co.* (USNM). *Moore Co.*: Southern Pines (USNM). *New Hanover Co.*: Wilmington (USNM). *Swain Co.*: 2.5 mi NNE Cherokee, GSMNP (SEMC); Andrews Bald, GSMNP (LSAM); Ekaneetlee Gap, GSMNP (LSAM). *Transylvania Co.*: Lake Toxaway (AMNH). *Watauga Co.*: 3 mi NW Blowing Rock (TAMU). *Yancey Co.*: Black Mountains (USNM, AMNH). **Ohio.** *Ashland Co.*: Mohican St. Pk. (FSC). *Champaign Co.*: Cedar Swamp (FSC). *Ottawa Co.*: Fishery Bay, S. Bass Isl. (CUIC). *Union Co.* (CUIC). **Oklahoma.** *Latimer Co.* (FSC, TAMU); 5 mi W Red Oak (CNC, TAMU). **Pennsylvania.** “Pen” (CNC, UASM). *Allegheny Co.*: Allegheny (CUIC). *Dauphin Co.*: Dauphin (CUIC). *Lehigh Co.*: Lehigh Gap (USNM). *Luzerne Co.*: Hazleton (MCZ). *Montgomery Co.*: Abington (MCZ). *Philadelphia Co.*: Frankford (USNM). *Pike Co.*: Twin Lakes (USNM). **South Carolina.** “S.C.” (MCZ). “Shiloh” (JCC). *Georgetown Co.*: Sandy Island (JCC). *Richland Co.*: Pontiac (JCC). **Tennessee.** “Chimney Camp, Gt. Smoky Mts.” (CUIC). *Blount Co.*: Cades Cove, GSMNP (LSAM, SEMC). *Cocke Co.*: Davenport Gap, GSMNP (LSAM). *Sevier Co.*: Goshen Prong, GSMNP (LSAM); Chimney Tops Picnic Nature Trail, GSMNP (LSAM); Roaring Fork, GSMNP (LSAM); Brushy Mnt., GSMNP (LSAM). **Texas.** *Brazos Co.*: College Station (TAMU). **Vermont.** *Bennington Co.*: Manchester (MCZ). *Chittenden Co.*: Burlington (USNM). *Orange Co.*: 12 mi E Chelsea (TAMU). **Virginia.** “Middletown” (MCZ). “Franklin Park” (USNM). *Arlington Co.*: Glencarlyn (USNM). *Fairfax Co.*: Vienna (USNM); Black Pond (USNM); Great Falls (USNM); Great Falls N.P. near Clay Pond (USNM); Great Falls N.P. near quarry site (USNM). *Giles Co.*: Mountain Lake, Univ. Va. Biological Sta. (USNM). *Lee Co.*: Pennington Gap (MCZ). *Loudoun Co.*: Middleburg (USNM). *Louisa Co.*: Gum Spring (USNM). *Montgomery Co.*: Blacksburg (CUIC). *Nelson Co.* (USNM). *Page Co.*: Skyland (CUIC, MCZ). *Rockbridge Co.*: Natural Bridge (USNM). *Shenandoah Co.*: New Market (USNM). *Warren Co.*: 7 km NNE Linden, summit of Blue Mt. (USNM). Alexandria (USNM). **West Virginia.** *Greenbrier Co.*: W. Sulphur (USNM). *Jefferson Co.*: Harpers Ferry (USNM); Shepherdstown (USNM). *Pocahontas Co.*: Cranberry Glades (USNM). *Preston Co.*: Aurora (USNM). **Wisconsin.** “Wis” (MCZ). *Bayfield Co.*: Bayfield (USNM). *Douglas Co.*: Bennett (USNM). *Sauk Co.*: Sauk City (GMNH). *Shawano Co.*: Tilleda (FSC). *Wood Co.*: Griffith State Nursery (USNM). **Wyoming.** *Weston Co.*: Newcastle (USNM).

#### Remarks.

This species varies in regard to the punctation and setae. The punctation on the pronotum is coarse and in most specimens free on the disc and very close, in part subcontiguous over the sides; in some specimens the punctation is denser, being subcontiguous on the disc and contiguous all over the lateral sides. The slanting setae on the elytra are usually semierect but in some specimens they are less inclined and the erect setae are difficult to distinguish. The erect setae are usually short and moderately numerous but in some specimens, they can be relatively long or much more numerous; in such case the species can be confused with *Paratenetus fuscus* but is easily separated by the coarse, irregular punctation on the pronotum and by the longer metaventrite.

Females are more common in collections than males. Of 220 randomly selected specimens, 169 (77%) were females and 51 (23%) were males.

Specimens were collected in March (n=6), April (n=89), May (n=296), June (n=384), July (n=152), August (n=67), September (n=40), October (n=9), November (n=5), and December (n=2).

Labels on specimens read “in overwintered nest remains of *Malacosoma americana* on *Prunus serotina* at mixed forest edge, shale barren area” (7 specimens), “shaken from and reared in moldy frass in old nest of *Malacosoma americana* on *Prunus serotina*” (13), “beaten from dead leaf clusters on cut branches of *Carpinus caroliniana* at forest edge” (6), “beaten from dead leaf clusters on branches of fallen *Populus deltoides*” (7), “beaten from dead leaf clusters on fallen broken branch of *Tilia americana* in shade, mixed forest” (2), “beaten from dead hanging leaf clusters on fallen *Ailanthus* in mixed forest” (6), “shaken from dead leaves on fallen branches of *Quercus rubra*” (4), “in moldy leaf clusters on fallen branch of *Quercus alba* in shade” (6), “beaten from dead leaves of wind-blown *Quercus rubra*” (1), “beaten from dead leaf clusters on fallen branches of *Quercus rubra* in mixed forest” (14); “at black light in longleaf pine and mixed oak, sand barrens” (23), “in moldy leaves on fallen branches of *Acer rubrum*” (4), “at black light in oak & longleaf pine sand barren” (5); “at black light; open sandy gap in mixed forest” (1); “at black light in mixed deciduous forest” (1); “at black light in mixed hardwood and loblolly pine forest” (1); “at black light in mixed pine and hardwood forest” (3); “beaten from dead leaf clusters on branches of *Castanea* out ca. 2 weeks earlier” (5); “at black light near mixed forest, farmed fields and tidal creek” (4); “beach drift” (1); “from pile of moldy thatch” (1); “in moldy leaf clusters on cut branches of *Prunus serotina*” (4); “in moldy leaf clusters on cut branches of *Morus*” (2); “beaten from dead leaf clusters on cut branches of *Acer rubrum* at mixed forest edge” (10); “in old nest of *Malacosoma* on *Prunus*” (2); “in dead leaves on branches of fallen oak” (1); “shaken from dead leaves on cut *Sassafras*” (7); “beaten from dead leaf clusters on fallen branch *Acer negundo* at mixed forest edge” (1); “at black light in tree canopy, mixed broken forest and residential area” (43); “at black light in mixed hardwood forest near pond and river” (8); “at black light in mixed forest, bluff above river” (2); “in old tent *Malacosoma americana*” (5); “at black light” (2); “in old tent nest of *Malacosoma americana* with moldy frass, on *Prunus serotina*” (1); “shaken from dry leaf (*Vitis* sp.) nest of *Sciurus carolinensis* in vine tangle ca. 3 m above ground” (1); “at black light at edge of clearing in mixed forest near drying vernal pool” (2); “at black light in mixed forest near vernal pools” (8); “at black light sheet in open mature mixed forest near river” (3); “beaten from dead leaf clusters on fallen branch of *Liriodendron* in mixed forest” (9); “beaten ex spruce” (1); “collected in tents *Malacosoma americana*” (12); “in web of *Malacosoma*” (3); “on *Pinus strobus*” (2); “ex. canopy trap” (34); “intercept trap” (1); “beating dead leaves” (8); “btng oak blowdown” (2); “leaf litter” (1); “dead moldy leaves” (1); “beating veg.” (2); “beating flowers” (2).

### Paratenetus exutus

Bousquet & Bouchard sp. n.

http://zoobank.org/E79EDDDF-59F8-4A43-880F-2A86CF8EB2F2

http://species-id.net/wiki/Paratenetus_exutus

Figs 1 , 2 , 7 , 13

#### Type material.

Holotype (♂) labeled “Tabusintac, N.S. 20-VI-1939 W.J. Brown / Holotype Paratenetus exutus Bousquet & Bouchard CNC No. 24035.” The specimen is deposited in the CNC.

Paratypes from the following localities: **Manitoba.** Ninette, 31-V-1958, J.F. McAlpine (2, CNC); same locality, 30-V-1958, R.B. Madge (1, CNC). **New Brunswick.** Tabusintac, 19-VI-1939, W.J. Brown (2, CNC); same data but 20-VI. 1939 or 22-VI-1939 (4, CNC). York Co., 14 km WSW of Tracy, S of Rt 646, 45.6741°N, 66.8161°W, 26 April-10 May 2010, R. Webster & C. MacKay coll. (2, RWC). York Co., 15 km W of Tracy off Rt. 645, 45.6848°N, 66.8821°W, 19–25 May 2009, R. Webster & M.-A. Giguère coll. (3, RWC). York Co., New Maryland Charters Settlement, 45.8430°N, 66.7275°W, 12 July 2005, R. P. Webster coll. (1, RWC); same locality but 45.8340°N, 66.7450°W, 30 April 2005 (1, RWC). Queens Co., Cranberry Lake P.N.A., 46.1125°N, 65.6075°W, 24 April-5 May 2009, R. Webster & M.-A. Giguère coll. (1, RWC); same locality but 3–13 May 2011, M. Roy & V. Webster coll. (1, RWC). Carleton Co., Jackson Falls, “Bell Forest”, 46.2200°N, 67.7231°W, 28.April-9 May 2009, R. Webster & M.-A. Giguère coll. (1, RWC). Carleton Co., Wakefield Meduxnekeag Valley Nature Preserve, 46.1890°N, 67.6766°W, 8 June 2005, M. Giguère & R. Webster coll. (1, RWC); same locality but 46.1935°N, 67.6825°W, 19 April 2995 (1, RWC). Albert Co., Shepody N.W.A., Germantown Section, 45.7101°N, 64.7542°W, 30 July 2004, R.P. Webster coll. (1, RWC). Sunbury Co., Acadia Research Forest, 45.9866°N, 66.3841°W, 8–13 May 2009, 13–19 May 2009, 19–25 May 2009, 16–24 June 2009, R. Webster & M.-A. Giguère coll. (10, RWC). **Nova Scotia.** St. Peters, 25-VII-1930, M.L. Prebble (1, CNC). **Ontario.** Alfred bog, 16.VI.1981, A. Davies (1, CNC). **Quebec.** Sainte-Catherine Portneuf, 29-VIII-1971, Claude Chantal (1, CNC). D[ivision de] R[ecensement] Bellechasse, St-Nérée, 10.VII.1976, J.F. Landry (3, CNC). Cascapedia, 11.VI.1933, W.J. Brown (1, CNC).

#### Etymology.

The specific name comes from the Latin participle *exutus*, -*a*, -*um* (deprived of) and alludes to the fact that the protibia of the male lacks the spinelike projection (calcar) found in the other American (north of Mexico) species.

#### Diagnosis.

This species is best separated from *Paratenetus punctatus* and *Paratenetus texanus* in having the antennomere 8 transverse. The males are also easily recognized among the species treated here in having no calcar on the protibia and a relatively long apical spine, oriented more or less parallel to long axis of tibia, on the mesotibia.

#### Description.

Body dorsally pale reddish brown in most specimens, with the pronotum and head usually slightly darker than elytra and legs; antennal club darker than antennomeres 1–8, particularly in males; metaventrite quite distinctly darker than first two abdominal ventrites in the vast majority of specimens, not or only slightly darker in a few specimens. Antennomere 8 transverse. Pronotum with maximum width at or very slightly anterior of midlength (Fig. 2); punctures narrowly spaced, in part subcontiguous over lateral half. Elytra less convex than for *Paratenetus gibbipennis* and *Paratenetus fuscus*; slanting setae subdepressed, erect setae few. Metaventrite long, length along midline longer than length of abdominal ventrite 2 along midline. Male protibia without calcar near middle along ventral surface; male mesotibia with relatively long, apical spine, oriented more or less parallel to long axis of tibia. Parameres with sides convergent towards apex; apex more or less truncate (Fig. 7).

Length: 2.5–3.0 mm.

**Figure 1.**
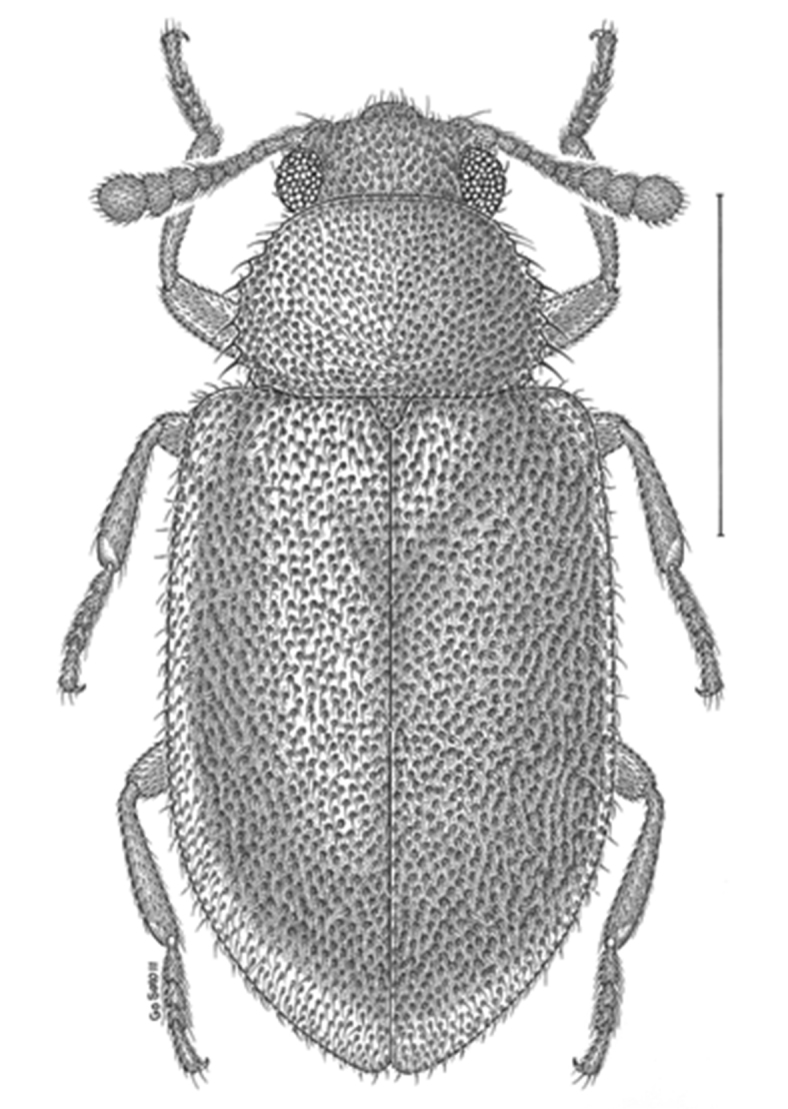
Dorsal habitus drawing of *Paratenetus exutus*.

**Figures 2–4.**
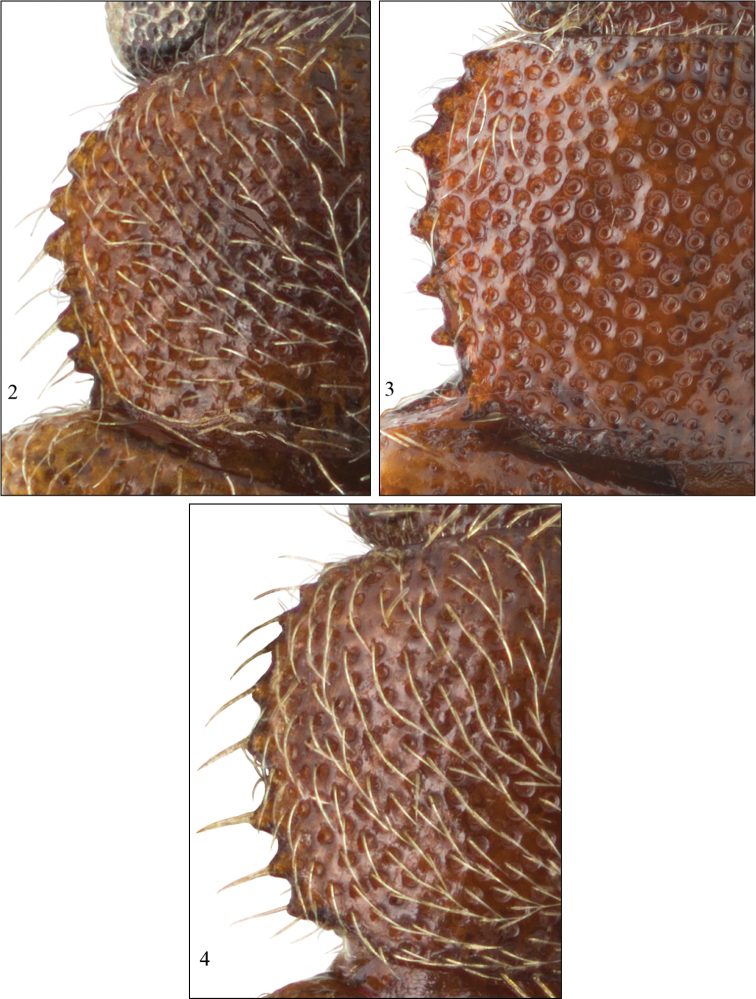
Left half of pronotum. **2** *Paratenetus exutus* **3** *Paratenetus punctatus* **4** *Paratenetus texanus*.

**Figures 5–9.**
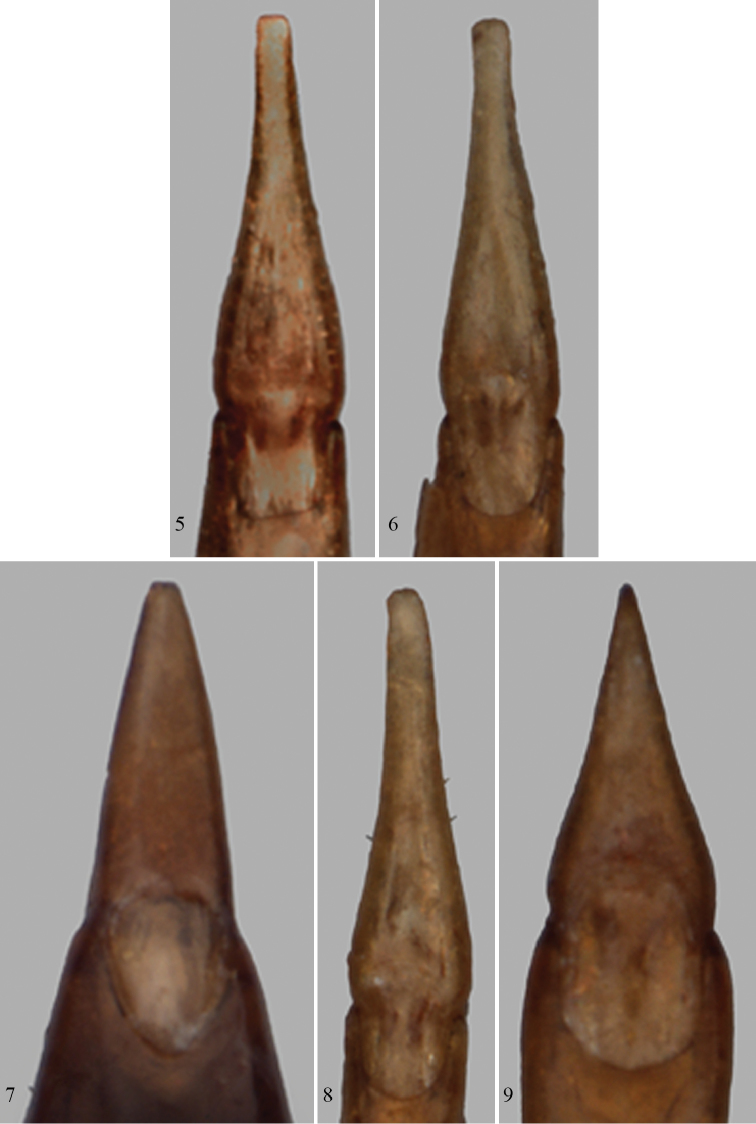
Parameres (dorsal view). **5** *Paratenetus gibbipennis* **6** *Paratenetus fuscus* **7** *Paratenetus exutus* **8** *Paratenetus punctatus* **9** *Paratenetus texanus*.

#### Distribution.

This species ranges from Cape Breton Island to northwestern Alberta, south to east-central Texas, southern Alabama, and southern Florida (Fig. 13).

**Figures 10–11.**
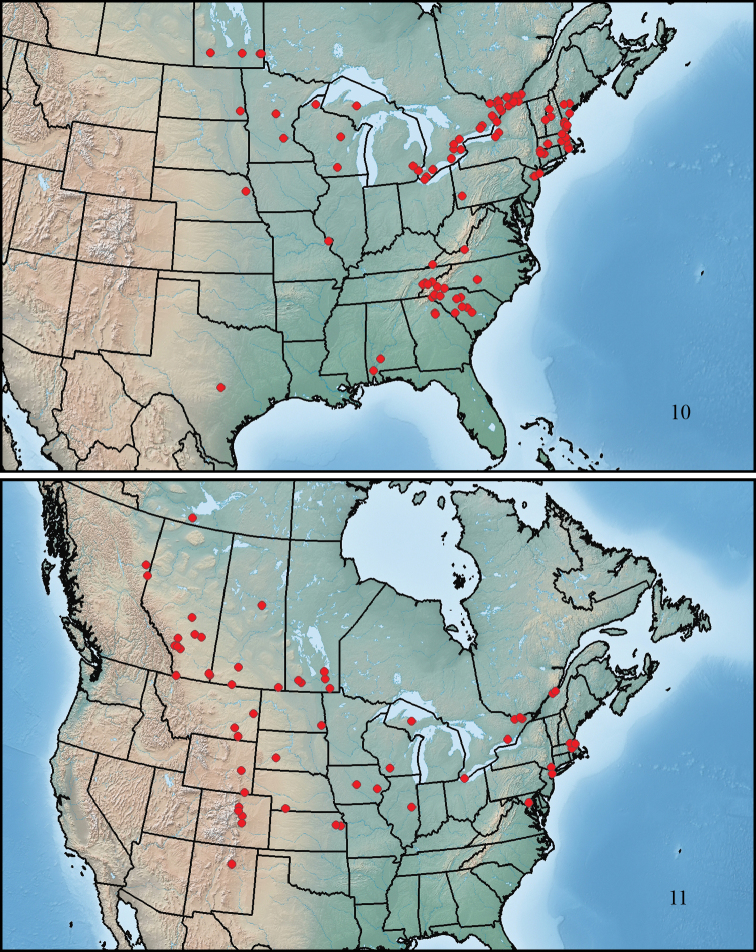
Maps showing collection localities in North America. **10** *Paratenetus gibbipennis* **11** *Paratenetus fuscus*.

**Figures 12–13.**
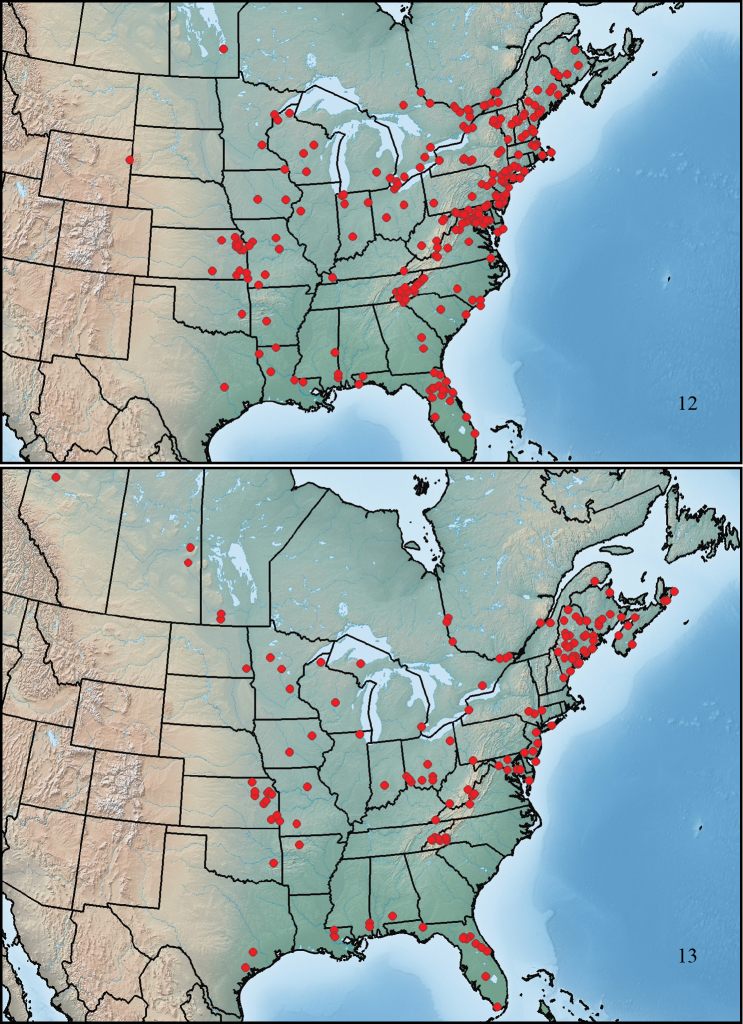
Maps showing collection localities in North America. **12** *Paratenetus punctatus* **13** *Paratenetus exutus*.

#### Records.

We have seen 416 specimens, including the type material, from the following localities. Canada. **Alberta.** “Tp. 73, Rge. 17, W. 4” (CNC). Peace River (NFC). **Manitoba.** Aweme (CNC). **New Brunswick.** Fredericton (CNC). **Nova Scotia.** Kentville (CNC). Annapolis Royal (CNC). Portapique (MCZ). St. Peter’s (AFC). Cape Breton (CNC, AFC). Grand River (CNC, AFC). Woodside (AFC). White Point Beach, Queens Co. (JCC). **Ontario.** Ridgeway (MCZ). Trenton (CNC). Prince Edward Co. (CNC). La Rose Forest, near Bourget (CNC). **Quebec.** Hull [= Gatineau] (CAS). Lac Duparquet (LEMM). Lac Labyrinthe [Abitibi] (LEMM). Laniel (CNC). Valcartier (CNC). **Saskatchewan.** Red Earth (RSM). Somme (RSM). United States of America. **Alabama.** *Conecuh Co.*: 19 km NE Evergreen (USNM). **Arkansas.** *Newton Co.*: 12 mi. W Jasper (SEMC). **Connecticut.** *Litchfield Co.*: Cornwall (RLAC, CUIC). **District of Columbia.** Washington (USNM). **Florida.** “Fla” (USNM). “Haulover” (USNM). *Alachua Co.*: Cross Creek (FSC); Gainesville (RLAC); nr. Paynes Prairie St. Pk. (FSC). *Brevard Co.*: Hatbill St. Pk. (FSC). *Dade Co.*: Everglades Nat. Pk. “Royal Palm Pk.” (CMN); Everglades Nat. Pk., Royal Palm Hammock (FSC). *Highlands Co.*: Archbold Biological Station (TAMU). *Lake Co.*: Camp McQuarrie (FSC). *Liberty Co.*: Torreya St. Pk. (FSC). *Putnam Co.*: 2 mi. SW Interlachen (FSC). *Volusia Co.*: Enterprise (USNM). **Illinois.** *Lake Co.*: Grayslake (SEMC). **Indiana.** *Monroe Co.*: Bloomington (FSC). **Iowa.** *Buchanan Co.*: Independence (USNM). *Polk Co.*: Walnut Woods St. Pk. (CUIC, USNM). **Kansas.** *Bourbon Co.*: 9 mi SW Ft. Scott (SEMC). *Crawford Co.*: 3 mi NE Pittsburg (SEMC). *Jefferson Co.*: 1 km SW Perry State Park (SEMC); University of Kansas Field Station, Nelson Ravine Forest (SEMC); The Falin Property, 1.5 km N jct. 94th St. & Kingman Rd. (SEMC). *Marshall Co.*: Alcove Springs State Park (SEMC). *Neosho Co.*: 2 mi SE Erie (SEMC). *Osage Co.*: Melvern Lake Project, Outlet Park (SEMC); Pomona Lake, Outlet Park (SEMC). *Pottawatomie Co.*: St. George (SEMC). *Wabaunsee Co.*: 10 mi SW Alma (SEMC). **Kentucky.** “Ky” (USNM). **Lousiana.** *East Baton Rouge Parish*: LA 37 at Comite River (LSAM). *East Feliciana Parish*: Boy Scout Camp Avondale, E of Clinton (LSAM); 1.2 mi S Central (LSAM). **Maine.** *Aroostook Co.*: St. Francis (DENH); Crystal (USNM); Howe Brook (USNM); Portage (USNM); Clayton Lake (USNM); Ashland (USNM). *Cumberland Co.*: South Portland (CUIC). *Franklin Co.*: Oquossoc (DENH). *Hancock Co.*: Blue Hill (DENH); E. Orland (USNM). *Kennebec Co.*: Vassalboro (USNM); Augusta (USNM). *Knox Co.*: Friendship (USNM). *Lincoln Co.*: New Harbor (USNM); Bristol (USNM); Boothbay Harbour (USNM). *Oxford Co.*: Peru (CUIC, MCZ). *Penobscot Co.*: Lee (USNM); Springfield (USNM). *Piscataquis Co.*: Kokadjo (DENH); Dover-Foxcropt (DENH); Chesuncook (USNM). *Somerset Co.*: Caratunk (DENH, USNM); Embden (USNM); Bingham (USNM); Brighton (DENH); Rockwood (USNM); Seboomook (DENH). *Waldo Co.*: Palermo (USNM). *Washington Co.*: Princeton (DENH, USNM); Wesley (DENH, USNM); Steuben (CNC). *York Co.*: West Lebanon (DENH). **Maryland.** *Carroll Co.*: Finksburg (USNM). *Somerset Co.*: Shelltown (USNM). *Talbot Co.*: Wittman (USNM); 3 km SE Easton (USNM). **Michigan.** *Marquette Co.*: Marquette (USNM). *Wayne Co.*: Detroit (USNM). **Minnesota.** *Becker Co.*: Itasca St. Pk. area (USNM). *Crow Wing Co.*: Lake Hubert (CNC). *Sherburne Co.*: Elk River (CNC). **Mississippi.** *George Co.*: Lucedale (CUIC). *Greene Co.*: Leakesville (CUIC). **Missouri.** *Greene Co.*: nr. James River (TAMU). *Randolph Co.*: 1 mi E Moberly (TAMU). **New Jersey.** *Atlantic Co.*: 5 mi. N Hammonton (RLAC). *Cape May Co.*: Anglesea (USNM). *Ocean Co.*: Lakehurst (CUIC). *Salem Co.*: Lake Hudson, near Deepwater (RLAC). *Union Co.*: Elizabeth (USNM). **New York.** *Suffolk Co.*: Yaphank, L.I. (USNM). *Ulster Co.*: West Park (CUIC); Slide Mt. (CUIC). **North Carolina.** *Buncombe Co.*: Oteen (USNM); 6 mi S Asheville (SEMC). *Haywood Co.*: 9 mi. W Waynesville (SEMC); Cataloochee, GSMNP (LSAM); Purchase Knob, GSMNP (LSAM). *Henderson Co.*: Fletcher (FSC). *Swain Co.*: Andrews Bald, GSMNP (LSAM); Clingmans Dome, GSMNP (LSAM). *Yancey Co.*: Black Mountains (AMNH). **North Dakota.** *Richland Co.*: Mirror Pool (USNM). **Ohio.** *Fairfield Co.*: Barnebey Center (RLAC). *Franklin Co.*: Worthington (RLAC). *Hamilton Co.*: Cincinnati (USNM). *Highland Co.* (FSC). *Hocking Co.*: Ward Township (RLAC). *Pike Co.*: Jackson Lake (RLAC). *Preble Co.*: Hueston Woods (RLAC). *Ross Co.*: Tar Hollow St. Pk. (FSC). *Trumbull Co.*: Phalanx (CUIC). **Oklahoma.** *Latimer Co.*: Red Oak (FSC, TAMU). **Pennsylvania.** *Fayette Co.*: 5 mi. W. Ohiopyle (USNM). **Tennessee.** *Cocke Co.*: Albright Grove (LSAM). *Sevier Co.*: Ramsey Cascade Trail, GSMNP (LSAM); Goshen Prong, GSMNP (LSAM); Indian Gap, GSMNP (LSAM). *Swain Co.*: near Charlies Bunion, GSMNP (FSC). **Texas.** *Colorado Co.*: Columbus (USNM). *Victoria Co.*: Victoria (USNM). **Virginia.** “Ft. Monroe” (USNM). Covington (FSC). *Bath Co.*: 9.6 km N Clifton Forge (CNC). *Lee Co.*: Pennington Gap (MCZ). *Loudoun Co.*: 3 km SE Lovettsville (USNM). *Montgomery Co.*: Caldwell Fields (FSC, TAMU). **West Virginia.** *Mingo Co.*: Justice (CUIC). *Pocahontas Co.*: Cranberry Glades (USNM). **Wisconsin.** *Bayfield Co.*: Bayfield (USNM). *Wood Co.*: Griffith State Nursery (USNM).

#### Remarks.

While almost all specimens from Canada and northern United States had the metaventrite distinctly darker than the first two abdominal ventrites, this is not the case with the specimens from the southern states. There is also variation in the width of the antennomere 8. Most specimens have that antennomere distinctly transverse, some specimens from the southern states (particularly Louisiana) have the antennomere 8 only slightly transverse.

Females are more common in collections than males. Of 105 randomly selected specimens, 76 (72%) were females and 29 (28%) were males.

Specimens were collected in March (n=9), April (n=38), May (n=84), June (n=58), July (n=79), August (n=40), September (n=22), October (n=5), November (n=3), and December (n=2).

Labels on specimens read “at black light near mixed forest, farmed fields and tidal creek” (4 specimens); “at black light at edge of mixed forest and open turf on hill” (1); “in moldy leaf clusters on cut branches of *Acer rubrum*” (3); “beaten ex spruce” (35); “beaten ex fir” (10); “on *Bumelia lanuginosa*” (1); “ex. spruce” (1); “ex. canopy trap” (15); “ex. FIT, near upper meadow” (1); “ex. FIT, near lower meadow” (3); “ex. canopy malaise, near lower meadow” (9); “ex. canopy FIT, near lower meadow” (3); “malaise trap” (6).

Most specimens of this species in collections are identified under the name “*Paratenetus inermis* Bsq. and Bouch.” since it was the intended name. Unfortunately, we realized that the name was already used by Champion only after the specimens were returned to their respective collections.

### Paratenetus texanus

Bousquet & Bouchard sp. n.

http://zoobank.org/E4FC7175-796F-4270-966D-93B4EE8E681A

http://species-id.net/wiki/Paratenetus_texanus

Figs 4 , 9 , 14

#### Type material.

Holotype (♂) labeled “Port Isabel, Tex. 20.X.1982 Lot 2 BF&JL Carr / Holotype Paratenetus texanus Bousquet & Bouchard CNC No. 24133.” The specimen is deposited in the CNC.

Paratypes from the following localities: **Texas.** Port Isabel, 17.X.1982, 20.X.1982, 30.III.1987, BF&JL Carr (6, CNC). 18 mi. E of Hebbronville, 25.III.1987, BF&JL Carr (10, CNC). Cameron Co., Brownsville, 19 July 1981, W.E. Steiner (2, USNM). Cameron Co., Palmito Hill Hist. Site, Hwy. 4 east of Brownsville, 12-X-1993, S.M. Clark (2, RLAC). Cameron Co., 11 mi. W Boca Chica, 28 Sept. 1976, R. Turnbow (3, FSC). Hidalgo Co., Mission, Bentsen State Park, 17 (or 18) July 1981, W.E. Steiner (2, USNM). Hidalgo Co., Anzalduas Co. Pk., 19 Oct. 1985, Wappes & Downie (2, FSC). Bee Co., Beeville, 19 June 1974, W.E. Steiner (1, USNM).

#### Etymology.

The specific name derives from the name of the state of Texas where the species has been commonly collected.

#### Diagnosis.

Members of this species can be distinguished from those of *Paratenetus punctatus* and *Paratenetus exutus* in having the punctures on the pronotum sparser, not subcontigous even on the lateral half. They can also be distinguished from most adults of *Paratenetus punctatus* by their smaller size and from most adults of *Paratenetus exutus* by the subquadrate antennomere 8 and metaventrite of same color as the first two abdominal ventrites.

#### Description.

Body dorsally yellow to pale reddish brown, with the pronotum and head usually slightly darker than elytra and legs; antennal club slightly darker than antennomeres 1–8 in many specimens, often reddish brown to partially piceous, yellowish and as pale as legs in some specimens; metaventrite not darker than first two abdominal ventrites. Antennomere 8 subquadrate. Pronotum with maximum width at midlength (Fig. 4); punctures moderately dense, not subcontiguous even over lateral half. Elytra less convex than *Paratenetus gibbipennis* and *Paratenetus fuscus*; slanting setae subdepressed, erect setae short. Metaventrite long, length along midline longer than length of abdominal ventrite 2 along midline. Male protibia with calcar near middle along ventral surface; male mesotibia with short, preapical spine, wide at base and oriented perpendicularly to long axis of tibia. Parameres with sides distinctly convergent towards apex; apex markedly acute (Fig. 9).

Length: 2.7–3.3 mm.

#### Distribution.

**Figure 14.**
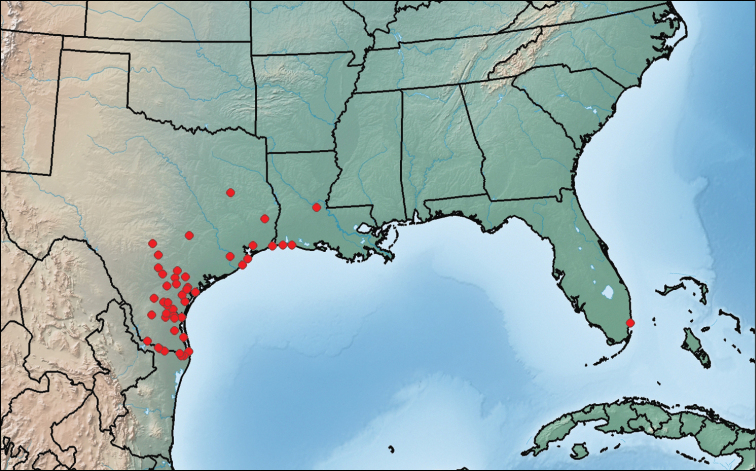
Map showing collection localities in America (north of Mexico) of *Paratenetus texanus*.

This species is known from southeastern Florida, central Louisiana, and central and eastern Texas (Fig. 14). We have also seen specimens from the states of Chiapas, Nayarit and Tamaulipas in Mexico.

#### Records.

We have seen 515 specimens, including the type material, from the following localities. United States of America. **Florida.** *Dade Co.*: Miami (FSC). **Louisiana.** *Avoyelles Parish*: Mansura (USNM). *Cameron Parish*: Holly Beach (LSAM, TAMU); nr. Oak Grove (TAMU). **Texas.** “60 mi SE Cotulla” (CNC). “15 mi SW Jct FR 3073 & Hwy 16” (CNC). *Anderson Co.*: Elkhart (TAMU). *Aransas Co.*: Goose Island St. Park (LSAM, TAMU). *Atascosa Co.*: Pleasanton (USNM); Campbellton (TAMU). *Bastrop Co.*: Bastrop St. Pk. (FSC). *Bee Co.*: Beeville (USNM); Pettus (CNC). *Bexar Co.*: San Antonio (USNM). *Brooks Co.*: Falfurrias (CNC); 9 mi W Falfurrias (TAMU). *Cameron Co.*: Boca Chica (CNC, TAMU); 6 mi W Boca Chica Beach (TAMU); 6.7 mi W Boca Chica Beach (TAMU); Brownsville (CNC, CUIC, MCZ, TAMU, USNM); 4 mi ESE Brownsville (TAMU); 6 mi E Brownsville (TAMU); 10 mi E Brownsville (RLAC, LSAM); 12.5 mi E Brownsville (TAMU); 13.5 mi E Brownsville (TAMU); W of Harlingen (TAMU); Main Reservoir near Brownsville (RLAC); Resaca de las Palomas St. Pk. (RLAC); Resaca de La Palma St. Pk. (TAMU); Sabal Palm Grove Wildlife Sanctuary (RLAC, GMNH, LSAM, TAMU); nr. Southmost (USNM); ca. 2 mi E Los Fresnos (TAMU); Laguna Atascosa NWR (TAMU); 9.7 mi E jct Rt 1419 on hwy 4 (TAMU). *Chambers Co.*: Anahuac (USNM). *Duval Co.*: San Diego (USNM); Freer (TAMU); Sepulveda Ranch (TAMU); 3.5 mi S Realitos (TAMU). *Fort Bend Co.*: Brazos Bend St. Pk. (TAMU). *Galveston Co.*: Virginia Point (USNM); San Luis Pass (TAMU); 3.5 mi SW Jamaica Beach (TAMU); 7 mi SW Jamaica Beach (TAMU). *Goliad Co.*: Goliad (USNM). *Hidalgo Co.*: Santa Ana Nat. Wdlf. Ref. (LSAM, TAMU, USNM); Bentsen Rio Grande Valley St. Pk. (LSAM, TAMU); Anzalduas Park (TAMU); Delta Lake (TAMU). *Jefferson Co.*: 10 mi W Sabine Pass (TAMU). *Jim Wells Co.*: Ben Bolt (CNC); 1 mi N Ben Bolt (TAMU); Alice (USNM); 5 km W Alice (CMN); 1 mi N Premont (TAMU); 1.4 mi S Premont (TAMU). *Karnes Co.*: 1 mi NE Runge (TAMU). *Kendall Co.*: Boerne (USNM). *Kenedy Co.*: Sarita (CNC); 2 mi S Sarita (TAMU); 13 mi S Sarita (TAMU); 25.3 mi S Sarita (FSC); 31.8 mi S Sarita (TAMU); Armstrong (CNC); 1 mi S Armstrong (TAMU); Norias (TAMU); 5 mi N Norias (TAMU); 6 mi S Norias (TAMU); 8 mi S Norias (CNC); Loyola Beach, Baffin Bay (CNC); Baffin Bay (TAMU). *Kleberg Co.*: Kingsville (CUIC, TAMU); Riviera (CNC, TAMU); Riviera Beach (CMN); Velederos Creek (TAMU). *Live Oak Co.*: 17 mi SW George West (TAMU). *Nueces Co.*: Corpus Christi (USNM, TAMU). *Refugio Co.*: 8 mi E Refugio (TAMU); 7 mi S Woodsboro (TAMU). *San Patricio Co.*: Sinton (USNM); nr. Sinton (CNC); 3 mi N Sinton (TAMU); 7 mi N Sinton (TAMU); Welder Wildlife Refuge (CMN, FSC, TAMU); Welder Wildlife Refuge, 17 km NE Sinton (CMN); Lake Corpus Christi St. Pk. (LSAM). *Starr Co.*: 1.5 m E Rio Grande City (LSAM). *Tyler Co.*: 4 mi E Spurger (TAMU). *Willacy Co.*: 8 miles SW Port Mansfield (TAMU). Mexico. **Chiapas.** El Aguacero, 16 km W Ocozocoautla (CMN); 5 km E Ocozocoautla (CMN); 2 km S Chicoasen (CMN); Cinco Cerros (CMN). **Nayarit.** 15 mi N Tepic (CNC). **Tamaulipas.** Mpio.San Carlos, Cerro del Diente (TAMU).

#### Remarks.

The two specimens from Miami in Florida externally agree perfectly with those from Texas. One is a male and its genitalia are identical to those of specimens from Texas.

Males are more common in collections than females. Of 106 randomly selected specimens, 42 (40%) were females and 64 (60%) were males.

Specimens were collected in January (n=1), February (n=1), March (n=65), April (n=39), May (n=89), June (n=30), July (n=53), August (n=21), September (n=36), October (n=108), November (n=2), and December (n=6).

Labels on specimens read “at black light in *Prosopis* and *Celtis* forest, sandy soil” (6 specimens); “on *Celtis*” (1); “ex dry okra pod” (1); “cotton” (1); “collected on *Celtis*” (2); “fallen fruit *Yucca treculeana*” (1); “on flower *Yucca treculeana*” (2); “on *Acacia Berlandieri* Benth.” (1).

This new species occurs in Mexico and nine species have been reported from that country. We have examined the type material of the six species described by Champion and housed in BMNH, i.e., *Paratenetus constrictus*, *Paratenetus corticarioides*, *Paratenetus nigricornis*, *Paratenetus punctulatus*, *Paratenetus tibialis*, and *Paratenetus villosus*, and none of them are conspecific with those of *Paratenetus texanus*. The three species not seen are *Paratenetus tropicalis* Motschulsky, *Paratenetus koltzei* Pic, and *Paratenetus mexicanus* Pic.

## Supplementary Material

XML Treatment for Paratenetus

XML Treatment for Paratenetus gibbipennis

XML Treatment for Paratenetus fuscus

XML Treatment for Paratenetus punctatus

XML Treatment for Paratenetus exutus

XML Treatment for Paratenetus texanus
